# Surgical treatment of post-inflammatory hydrocephalus. Analysis of 101 cases

**DOI:** 10.1007/s00381-018-4022-4

**Published:** 2018-12-18

**Authors:** Bartosz Polis, Lech Polis, Emilia Nowosławska

**Affiliations:** Department of Neurosurgery and Neurotraumatology, Polish Mothers Memorial Hospital Research Institute, 281/289 Rzgowska street, 93-338 Lodz, Poland

**Keywords:** Hydrocephalus, Post-inflammatory, Multiloculated, Shunt, Neuroendoscopy, NOSIC

## Abstract

**Objective:**

The aim of this paper was to evaluate the outcomes of surgical treatment for post-inflammatory hydrocephalus in pediatric patients. The patient’s age, surgical technique and type of implants, revision rate (depending on the cause for revision and shunt type), and final outcome measured with Neurologic Outcome Scale for Infants and Children (NOSIC) scale were evaluated.

**Methods:**

We performed a retrospective analysis of 101 patients with post-inflammatory hydrocephalus, treated in Polish Mother’s Memorial Hospital Research Institute since 2005. Children with comorbidities (e.g., tumors or hemorrhages) were excluded from the study. The assessment included patient age, surgical technique (ventriculoperitoneal shunt (VPS) or neuroendoscopy) and type of implant, revision rate (considering its cause), and final outcome measured in Neurologic Outcome Scale for Infants and Children (NOSIC) scale.

**Results:**

VPS implantation was the most common surgical technique. It was performed in 66.33% (*n* = 67) of cases. Neuroendoscopic procedure was used in 33.66% of cases (*n* = 34). Revision rate of VPS was 52.23% (*n* = 35). Endoscopic third ventricle ventriculostomy (ETV) was efficient only in 5 cases (14.7%), whereas in 29 cases (85.3%), it was followed by VPS implantation. Revision rate in VPS implantation after ETV reached 55.17% (*n* = 16). In all age groups, VPS implantation was the most frequently used procedure. Revisions of the shunt systems occurred most frequently in the 1–3 (*n* = 21 41.18%) and < 1 (*n* = 12, 23.53%) age ranges. The type of valve that most often underwent dysfunction was flow-regulated type (*n* = 23, 62.16%). The type of valve that was the least frequently revised was differential pressure type (*n* = 11, 17.18%). In all age groups, mechanical dysfunction was the most frequent cause of shunt disability. Average NOSIC score ranged from 39 to 98 (average 80.58, standard deviation ± 13.34). NOSIC result relative to individual operational techniques was as follows: ETV + VPS—80.17 (*n* = 29, standard deviation ± 11.44), VPS—80.44 (*n* = 67, standard deviation ± 14.30), and ETV—80.80 (*n* = 5, standard deviation ± 11.62). There was no difference between the outcome of the NOSIC and the type of implanted valve or its dysfunction.

**Conclusions:**

In our analysis, post-inflammatory hydrocephalus accounts for 11.7% of all hydrocephalus types. Of post-inflammatory hydrocephalus, multiloculated type accounts for 14.9%. The most common type of surgery in these patients is implantation of the ventriculoperitoneal system. The most frequent revisions of the VPS system occur in the group of the younger children (< 3). The most common type of a dysfunction shunt is the differential pressure valve, and the rarest type the flow-regulated type. In the case of mechanical dysfunction, occlusion of the intraventricular catheter is the most common reason. ETV does not affect the frequency of VPS revisions. The average NOSIC score in children treated with hydrocephalus is below normal, and the best results are observed in the youngest children.

## Introduction

Hydrocephalus is a relatively common disorder of the central nervous system (CNS) in pediatric patients. It affects 0.36–0.75 per 1000 live births [30, 33]. Hydrocephalus may be caused by multiple pathological conditions, including central nervous system (CNS) inflammation [1, 6, 19, 21, 23]. Post-inflammatory hydrocephalus is characterized by major disturbances in CSF circulation, sometimes forming obstructive blockages in the ventricular system. They may evolve into the multiloculated type of post-inflammatory hydrocephalus. Data regarding the incidence of post-inflammatory hydrocephalus vary widely in the world literature. Amacher reported 7.6%, Handler 30–40%, Ammirati 8%, and Rupprecht 4.6% [2, 3, 9, 20]. Australian data reported an incidence of 2.8% and those from Taiwan 31.25%. In Poland, the estimated range is 11–30%. Data concerning post-inflammatory hydrocephalus are sparse and based on small groups. Very often they show wide variations. The most common surgical procedure in hydrocephalus is the implantation of ventriculoperitoneal shunt (VPS) or endoscopic third ventricle ventriculostomy (ETV) [4, 5, 8, 11, 12]. The impaired development prognosis concerns the group of children with post-inflammatory hydrocephalus [22].

## Material and methods

We conducted a retrospective analysis of 101 patients with post-inflammatory hydrocephalus. Between 2005 and 2016, 864 children were operated for hydrocephalus in the Department of Neurosurgery of the Polish Mother’s Memorial Hospital Research Institute. Post-inflammatory hydrocephalus (PIH) was diagnosed in 101 cases. All PIH cases were categorized according to type of hydrocephalus, patient age, and time of surgery. All etiological factors of post-inflammatory hydrocephalus were identified. The microbiological aspect of post-infectious hydrocephalus was not analyzed in this paper. All patients underwent surgery. The follow-up period ranged from 6 to 12 months (mean 9.5 ± 3 months). All patients qualified for the analysis were originally treated in our center. Children with comorbidities (e.g., tumors or hemorrhages) were excluded from the study. The patients were divided into five groups depending on the time of surgical treatment: newborns up to 30 days of age (< 0 years), neonates and infants (30 days–1 year), younger children (1–3 years), pre-school children (3–6 years), and older children (> 6 years). Two aspects must be addressed in the treatment of post-inflammatory hydrocephalus: first, the treatment of any identified etiological factor(s), and second, the surgical treatment of hydrocephalus. With three consecutive CSF sterile cultures, CSF protein level ≤ 100 mg%, and pleocytosis not exceeding 100/μl, VPS was implanted as a method of choice in all cases of communicating hydrocephalus. In all cases in which physiological CSF circulation pathways were clearly obliterated, with diagnostic imaging showing evidence of non-communicating hydrocephalus (mono-, tri-, or quadriventricular), a neuroendoscopic procedure (ETV or septostomy) was implemented. It is possible that the ineffectiveness of these treatments will appear later. In cases mentioned above, the shunt could not be implanted because of abnormal CSF parameters or lack of sterile sample. All surgical procedures were performed under general anesthesia. The types of VPS used were as follows: programmable (*n* = 0), differential pressure (*n* = 64), and flow-regulated (*n* = 37). Silicon ventricular and peritoneal catheters impregnated with 0.15% clindamycin and 0.54% rifampicin were used [32]. The selection of shunt type was made at the discretion of the operator. We assessed the number of revision surgical procedures and VPS dysfunctions according to the type of valve used and the age of the patient. The final outcome was measured using the NOSIC scale. The following domains of neurologic function were included: general level of consciousness, current mental status (according to age-appropriate measures), motor function, visual attention, speech and hearing, learning and schooling, task attention, social disinhibition, activities of daily living, and the caretakers’ perception of the impact the child’s neurologic status on his/her activities. Scoring of items in the NOSIC scale includes adjustment for items that cannot be assessed in individual cases. PGOS-E and PCPS scales were not used because they do not account for the developmental level of the child and were created primarily for the assessment of brain traumatic injury [10, 15–18, 31]. The range 84–100 was assumed to be the normal value. Parametric data were presented as arithmetic mean and standard deviation. For larger group numbers, ANOVA variance analysis was used. The statistical hypothesis was verified at *p* ≤ 0.05 level. All calculations were performed with Statistica 10.0 software.

## Results

**Fig. 1:**
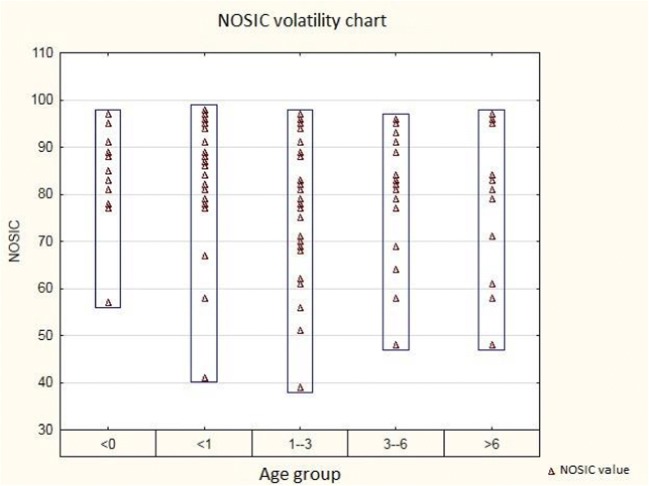
NOSIC volatility in individual age groups

The analyzed group consisted of 101 cases of post-inflammatory hydrocephalus. Post-inflammatory hydrocephalus was diagnosed in 11.7% (*n* = 101) of all hydrocephalic patients (*n* = 864). Multiloculated hydrocephalus was diagnosed in 14.9% (*n* = 15) cases. The patients’ age ranged from 0 to 16 years (mean 2.81 years, standard deviation ± 3.69). Male to female ratio was 1.13:1 (*n* = 53♂:*n* = 47♀). The analyzed group was divided into 5 age subgroups (age at the time of surgery): < 0 years, 10.89% (*n* = 11); 0–1 year, 22.77% (*n* = 23); 1–3 years, 38.61% (*n* = 39); 3–6 years, 14.85% (*n* = 15); and > 6 years, 12.87% (*n* = 13) [Table 1]. Primary ventriculoperitoneal shunt implantation was the most common surgical technique. It was performed in 66.33% of cases (*n* = 67). The neuroendoscopic procedure was used in 33.66% of cases (*n* = 34). The revision rate of VPS was 52.23% (*n* = 35). ETV was efficient only in 5 cases (14.70%), and in 29 cases (85.29%), it was followed by VPS implantation. The revision rate for post-ETV VPS implantation reached 55.17% (*n* = 16). The frequency of revision of the VPS system in patients initially treated with VPS and in those initially treated with ETV followed by VPS implantation did not differ significantly (*p* = 0.8715). Two types of shunt mechanisms were used: the differential pressure type (*n* = 64, 63.36%) and the flow-regulated type (*n* = 37, 36.63%). The programmable valve was not used in any of the analyzed cases. Primary VPS implantation was the most frequently used procedure in all age groups (< 0 years, *n* = 6, 54.55%; < 1 year, *n* = 14, 60.83%; 1–3 years, *n* = 26, 66.67%; 3–6 years, *n* = 10, 66.67%; and > 6 years, *n* = 11, 84.62%; *p* = 0.1299). In addition, the number of patients requiring post-ETV VPS implantation in consecutive age groups was as follows: < 0 years, *n* = 3, 27.27%; < 1 year, *n* = 6, 26.08%; 1–3 years, *n* = 13, 33.33%; 3–6 years, *n* = 5, 33.33%; > 6 years, *n* = 2, 15.38%. Effective ETV (or septostomy) without subsequent VPS implantation was recorded only in the following age groups: < 0 years, *n* = 2, 18.18%; and < 1 year, *n* = 3, 13.04%. Shunt system revisions following primary implantation or implantation after unsuccessful ETV (or septostomy) occurred most frequently in the < 1-year (*n* = 12, 60%) and 1–3-year (*n* = 21 53.84%) age ranges (*p* = 0.6671). The types of valve used most commonly for both primary and post-ETV procedure in individual age categories were as follows: < 0 years (differential pressure, *n* = 6, 66.66%), < 1 year (differential pressure, *n* = 12, 60%), 1–3 years (differential pressure, *n* = 28, 71.79%), 3–6 years (differential pressure, *n* = 10, 66.66%), and > 6 years (differential pressure, *n* = 8, 61.53%) *p* = 0.3747. The type of valve with the highest rate of dysfunction was the differential pressure type (*n* = 44, 45.83%), *p* = 0.0017. The least frequently revised valve was the flow-regulated type (*n* = 23, 23.95%) (*p* = 0.0017). In all age groups, mechanical dysfunction was the most common cause of shunt failure due to ventricular catheter obstruction (< 0 years, *n* = 3, 27.27%; < 1 year, *n* = 8, 34.78%; 1–3 years, *n* = 17, 43.59%; 3–6 years, *n* = 5, 33.33%; > 6 years, *n* = 7, 53.85%; *p* = 0.9633). Infection of the shunt system (*n* = 3, 13.04%) and its overdrainage (*n* = 1, 4.35%) occurred most often in the < 1-year age group (*p* = 0.9633). All recognized cases of overdrainage were noted only in flow-regulated shunt receivers aged < 1 year (*n* = 1) and 1–3 years (*n* = 1). In both cases, there was a massive enlargement of the ventricular system. In 55.56% of cases, infection occurred in the flow-regulated type of shunt (*n* = 9). A mechanical cause of VPS dysfunction was identified in 24 cases with differential pressure shunts and in 16 cases with flow-regulated shunts (*p* = 0.1372). The average NOSIC scores ranged from 39 to 98 (mean 80.58, standard deviation ± 13.34). The average distribution of NOSIC results per age group was as follows: < 0 years—83.27 (*n* = 11, standard deviation ± 10.97), < 1 year—83.17 (*n* = 23, standard deviation ± 13.25), 1–3 years—79.10 (*n* = 39, standard deviation ± 13.17), 3–6 years—79.26 (*n* = 15, standard deviation ± 14.06), and > 6 years—79.30 (*n* = 13, standard deviation 15.74). Detailed NOSIC distribution is shown in Fig. 1.

**Table 1:** Types of surgical procedures in individual age groups

Age group	< 0				
Procedure:		Number	%	Revision	%
ETV (inc septostomy)		2	18.18	0	0
ETV + VPS		3	27.27	1	33.33
VPS		6	54.54	3	50
*Ʃ*		11			
Age group	< 1				
Procedure:		No.	%	Rev.	%
ETV (inc septostomy)		3	13.04	0	0
ETV + VPS		6	26.08	5	83.33
VPS		14	60.86	7	50
*Ʃ*		23			
Age group	1–3				
Procedure:		No.	%	Rev.	%
ETV (inc septostomy)		0	0	0	0
ETV + VPS		13	33.33	8	61.53
VPS		26	66.66	13	50
*Ʃ*		39			
Age group	3–6				
Procedure:		No.	%	Rev.	%
ETV (inc septostomy)		0	0	0	0
ETV + VPS		5	0	0	0
VPS		10		6	60
*Ʃ*		15			
Age group	> 6				
Procedure:		No.	%	Rev.	%
ETV (inc septostomy)		0	0	0	0
ETV + VPS		2	15.38	2	100
VPS		11	84.61	6	54.54
*Ʃ*		13

**Fig. 2:**
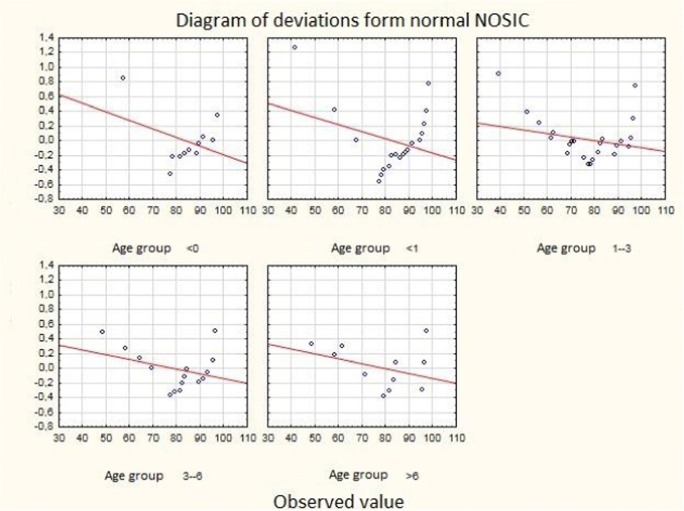
Diagram of deviations from normal NOSIC in individual age groups

**Fig. 3:**
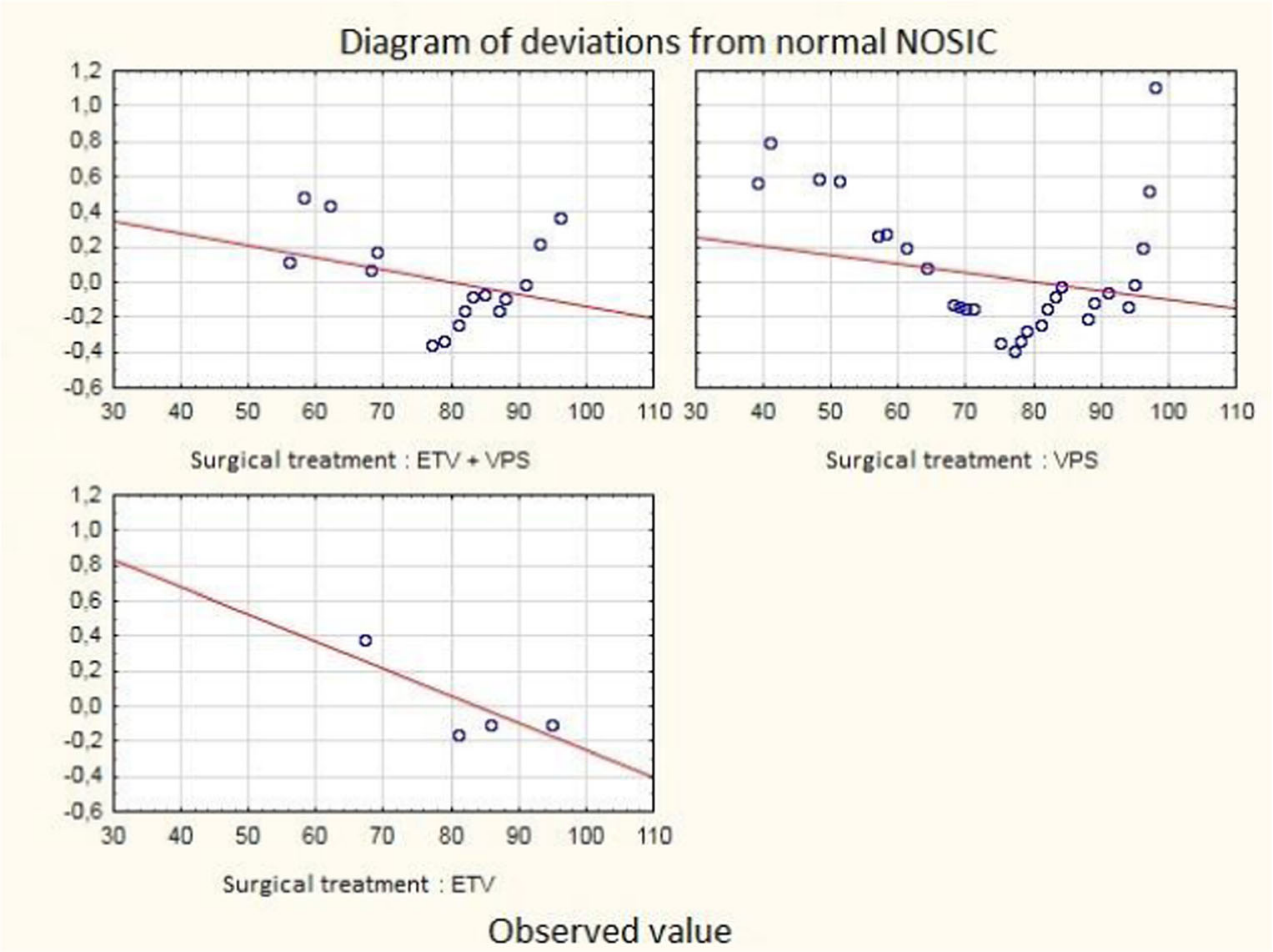
Diagram of deviations from normal NOSIC regarding the type of surgery

The best NOSIC results were observed in two youngest age groups (no statistical significance). Deviations from the normal NOSIC score in individual age groups are shown in Fig. 2. The NOSIC score depending on individual operational techniques was as follows: ETV + VPS—80.17 (*n* = 29, standard deviation ± 11.44), VPS—80.44 (*n* = 67, standard deviation ± 14.30), and ETV—80.80 (*n* = 5, standard deviation ± 11.62). A detailed distribution of NOSIC scores with respect to the operating technique is shown in Fig. 3. There was no difference in the NOSIC score relating to the type of implanted valve or its dysfunction.

## Discussion

Post-inflammatory hydrocephalus is a severe and life-threatening disease. Data regarding its incidence are scarce and vary significantly. In developed countries, disease-related mortality rate is up to 10%, and neurological damage may occur in 30% of cases. In poor countries, these percentages are 50 and 60%, respectively [24]. Statistics on post-inflammatory hydrocephalus vary widely. Discrepancies regarding its incidence range from 4.6 to 40% [2, 3, 20]. About 80% of cases occur in children before the age of 5 years. Generally, bacterial meningitis is the result of blood-brain infection. The causative pathogens can usually be traced back to the mucous membranes. Through blood, microorganisms penetrate into the intracranial subarachnoid space. They cross the blood-brain barrier via choroid plexuses, cerebral capillaries, or arachnoid dura. The passage of microorganisms through the blood-brain barrier, their multiplication, and release of toxins triggers a cascade of inflammatory processes. Inflammation destroys the bacteria, a process that can be facilitated by administration of antibiotics. Microbial breakdown products damage the vascular endothelium with subsequent disruption of the blood-cerebrospinal fluid barrier. As a result, low molecular weight serum proteins, electrolytes, and water leak through the vascular wall to the surrounding tissue, leading to cerebrovascular edema. At a later stage, inflammatory factors trigger the inflow into the subarachnoid space of neutrophilic granulocytes whose products (fatty acid metabolites and free radicals) cause cytotoxic edema. The result is a further increase in intracranial pressure (ICP) with consequent reduction in cerebral blood flow. Meningitis can be associated with various forms of hydrocephalus. Impaired CSF flow may result from the narrowing of fluid pathways by inflammatory effusion, adhesions of opposite surfaces of intravascular lining, further aggravated by cerebral edema. Post-inflammatory narrowing of the Monro foramen can lead to unilateral widening of the lateral ventricle. Impaired flow through the cerebral aqueduct causes triventricular hydrocephalus. Tetraventricular hydrocephalus occurs when the obstruction is located at the level of the central and lateral foramina in the roof of the fourth ventricle. According to the classic division, this type of hydrocephalus is described as non-communicating [26, 27]. The purulent exudate in the subarachnoid vault and basal cisterns damages the arachnoid villi leading to impaired CSF absorption. The result is the widening of the entire ventricular system and subarachnoid space. This condition is referred to as communicating hydrocephalus [26, 27, 29]. Three methods are used for routine imaging of hydrocephalus: cranial ultrasound (cUSG), CT, and NMR. Ultrasonography is used in patients with open anterior fontanelle. Prior to the neurosurgical intervention, each patient underwent cranial CT or NMR examination. The treatment of post-inflammatory hydrocephalus is bimodal, including procedures that address excessive CSF retention and those directed against the causative agent. The clinical symptoms of intracranial hypertension and widening of fluid spaces in imaging studies guide the selection of appropriate methods of temporary removal of excess CSF. These include lumbar punctures (communicating hydrocephalus), implantation of subcutaneous ventricular access (Rickham’s reservoir), or implantation of external ventricular drainage. The above procedures provide decompression of the ventricular system, reduce the risk of inflammation spreading outside the CNS, and help treat nervous system infections. The situation is different if excessive CSF retention occurs after the inflammation has resolved. The basic method of treatment is drainage using a VPS system (for communicating hydrocephalus) or neuroendoscopic surgery (for non-communicating hydrocephalus). The VPS system is the most common according to both literature data and those in our material. It was used in 66.33% of cases presented in this paper. Neuroendoscopic surgery for either non-communicating hydrocephalus or multiloculated hydrocephalus was performed in 33.6% of cases. Unfortunately, ETV as the sole therapeutic method was effective only in 14.7% of cases. All the remaining cases of ETV required subsequent valve implantation. The highest effectiveness of neuroendoscopic procedures in post-infectious hydrocephalus can be explained by differences in hydrodynamics of CSF circulation in children aged < 2 years. In this age group, CSF absorption may occur in the perivascular spaces of venules. It is possible that the ineffectiveness of these treatments will appear in the longer term. The percentage of VPS revisions in patients treated a priori with the VPS system was 52.23%. However, the percentage of shunt system revisions in patients with post-ETV placements of VPS was 55.17%. The most commonly used type of shunt was the differential pressure shunt. The highest frequency of shunt dysfunction occurred with the differential pressure valve, and the lowest with the flow-regulated valve. VPS system revision was the most common in the 1–3-year age group. In all age groups, mechanical failure (occluded intraventricular catheter) was the most frequent cause of VPS dysfunction. Although the majority of children with hydrocephalus are likely to have a healthy development, it is estimated that 19.5–25% of patients experience increased difficulties leading to developmental delay at least to a slight extent [7]. Psychological research conducted in various centers indicates that the basic characteristic of cognitive functioning of children with hydrocephalus is disharmonious development of the verbal zone and executive functions [7, 13, 14]. This trend is also apparent in those children with hydrocephalus whose global level of cognitive development is within the normal range [34]. Irrespective of the general level of intellectual development, disorders in the development of memory functions, especially non-verbal memory, tend to be more frequent in children with hydrocephalus. Selective disorders in the development of basic cognitive processes are also accompanied by behavioral changes, such as apathy and decreased interest in the environment, slowing down of thought processes as well as disturbances in the development of the emotional and personality zone [7, 25]. There is lack of consistency in the literature regarding the impact of hydrocephalus etiology on the degree of mental impairment. According to D. Riva and colleagues, neither the etiology of hydrocephalus nor the duration of intracranial hypertension seems to have an effect on cognitive development [28]. However, according to research by J. Hirsh, the worst prognosis is expected for children with post-inflammatory hydrocephalus [22]. In the study group, the mean point value of NOSIC was 80.58, which is below the standardized limit of normal. In the two youngest age groups, the test results appear to be better than those in the other groups. The type of surgical technique or valve, as well as the presence or absence of shunt dysfunction, had no impact on the final NOSIC score.

## Conclusions

In our analysis, post-inflammatory hydrocephalus accounted for 11.7% of all types of hydrocephalus. In the group of post-inflammatory hydrocephalus, the multiloculated type constituted 14.9% of cases. The most common type of surgery in these patients was the implantation of ventriculoperitoneal shunt system. The highest VPS system revision rates were noted in the younger children age group (< 3 years). The type of shunt most likely to become dysfunctional was the differential pressure valve, and the least likely was the flow-regulated valve. Occlusion of the intraventricular catheter was the most common cause of mechanical dysfunction. No difference was observed in the number of VPS revisions in patients who were initially treated with VPS and those treated with ETV followed by VPS implantation. The average NOSIC score in children treated for hydrocephalus was below normal, and the best results were observed in the youngest children.
